# Intraosseous intraneural perineurioma derived from the inferior alveolar nerve with an abnormality of chromosome 22 and expression of the BCR-ABL fusion gene: report of a case and review of recent literature

**DOI:** 10.1186/s12957-018-1481-8

**Published:** 2018-09-13

**Authors:** Jun Kurihara, Satoshi Yokoo, Miku Ichikawa, Takahiro Shimizu, Masaru Ogawa, Mai Seki

**Affiliations:** 1 Department of Oral and Maxillofacial Surgery, and Plastic Surgery, Gunma University Graduate School of Medicine, 4-39-15 Showa-machi, Maebashi, Gunma 371-8511 Japan; 2 Department of Pathological Diagnostics, Gunma University Graduate School of Medicine, 4-39-15 Showa-machi, Maebashi, Gunma 371-8511 Japan

**Keywords:** Perineurioma, Chromosome 22 abnormality, BCR-ABL fusion gene, INPN, ENPN

## Abstract

**Background:**

Perineurioma (PN) is a peripheral nerve disease that primarily develops in the limbs and trunk and very rarely occurs in the oral cavity. PN is classified into two types: intraneural perineurioma (INPN) and soft tissue perineurioma (extraneural perineurioma, ENPN). In this article, we report a patient with mandibular body INPN derived from the perineurium of the inferior alveolar nerve.

**Case presentation:**

The patient was a 43-year-old male. He consulted our department for a detailed examination of the right mandibular body. A biopsy was performed at another hospital and he was diagnosed with a schwannoma. At his first visit, hypesthesia extending from the right lower lip to the mental region was recognized and enlargement of the right mandibular canal was confirmed with X-ray CT and MRI. Considering the possibility of future tumor growth, we extirpated the tumor under general anesthesia. Cystic tumor was seen continuously in the inferior alveolar nerve. Immunohistologically, the tumor cells were positive for Glut-1, weakly positive for EMA, and weakly positive for Claudin-1, and the histopathological diagnosis was INPN. In addition, absence of the BCR region of chromosome 22 and expression of the BCR-ABL fusion gene were observed by fluorescent in situ hybridization (FISH), and a chromosome 22 abnormality was confirmed. These findings indicated that the disease was a neoplastic lesion.

**Conclusion:**

Expression of the BCR-ABL fusion gene in INPN that develops in the oral cavity is thought to be very rare, and to the best of our knowledge, ours is the first case to be reported in the literature. About three postoperative years have passed, but findings suggestive of recurrence have not been observed.

## Background

Perineurioma (PN) is a peripheral nerve disease that primarily develops in the limbs and trunk. Development in the oral cavity is rare. PN is classified into two types: intraneural perineurioma (INPN) and soft tissue perineurioma (extraneural perineurioma, ENPN). The incidence of INPN is lower than that of ENPN. A search of PubMed from 1981 until 2017 revealed only four case reports on mandibular INPN, including our report [1–3]. In addition, to the best of our knowledge, this is the first report on INPN of the inferior alveolar nerve with a chromosome 22 abnormality and BCR-ABL fusion gene expression, simultaneously. In this article, we report a patient with INPN in the mandibular body region derived from the perineurium of the inferior alveolar nerve and review the literature to analyze the pathogenesis.

## Case presentation

A 43-years-old male of Japanese Brazilian with hypesthesia from the right lower lip to the mental region consulted a hospital for the first visit. Computed tomography (CT) revealed a round radiolucent area in the right mandibular body, and biopsy was carried out, leading to a diagnosis of schwannoma. He was referred to the Department of Oral and Maxillofacial Surgery, Gunma University Hospital, for detailed examination and treatment for the first time in March 2015.

**Fig. 1:**
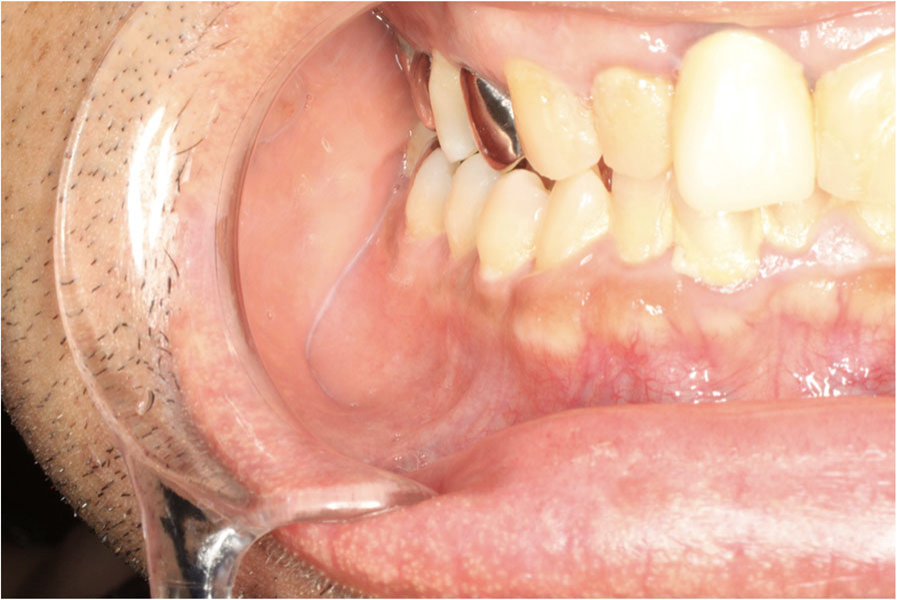
Intraoral findings. Although no obvious neoplastic lesions were observed, hypesthesia extending from the right lower lip to the mental region was noted

**Fig. 2:**
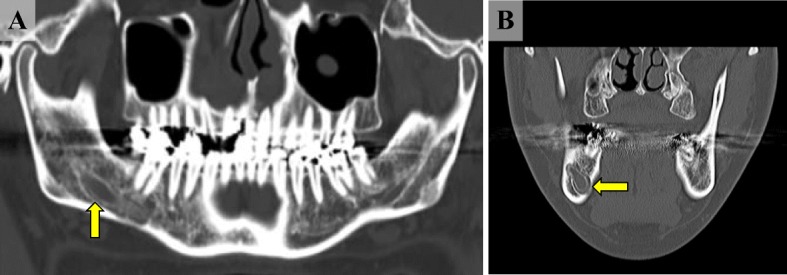
X-ray findings. **a** X-ray CT finding (panoramic image). A radiolucent finding of a similar circle was recognized in the right mandible body (yellow arrow). **b** X-ray CT finding (coronal image). We confirmed the enlargement of the right mandible canal. The destruction of the mandibular canal wall is not clear (yellow arrow)

**Fig. 3:**
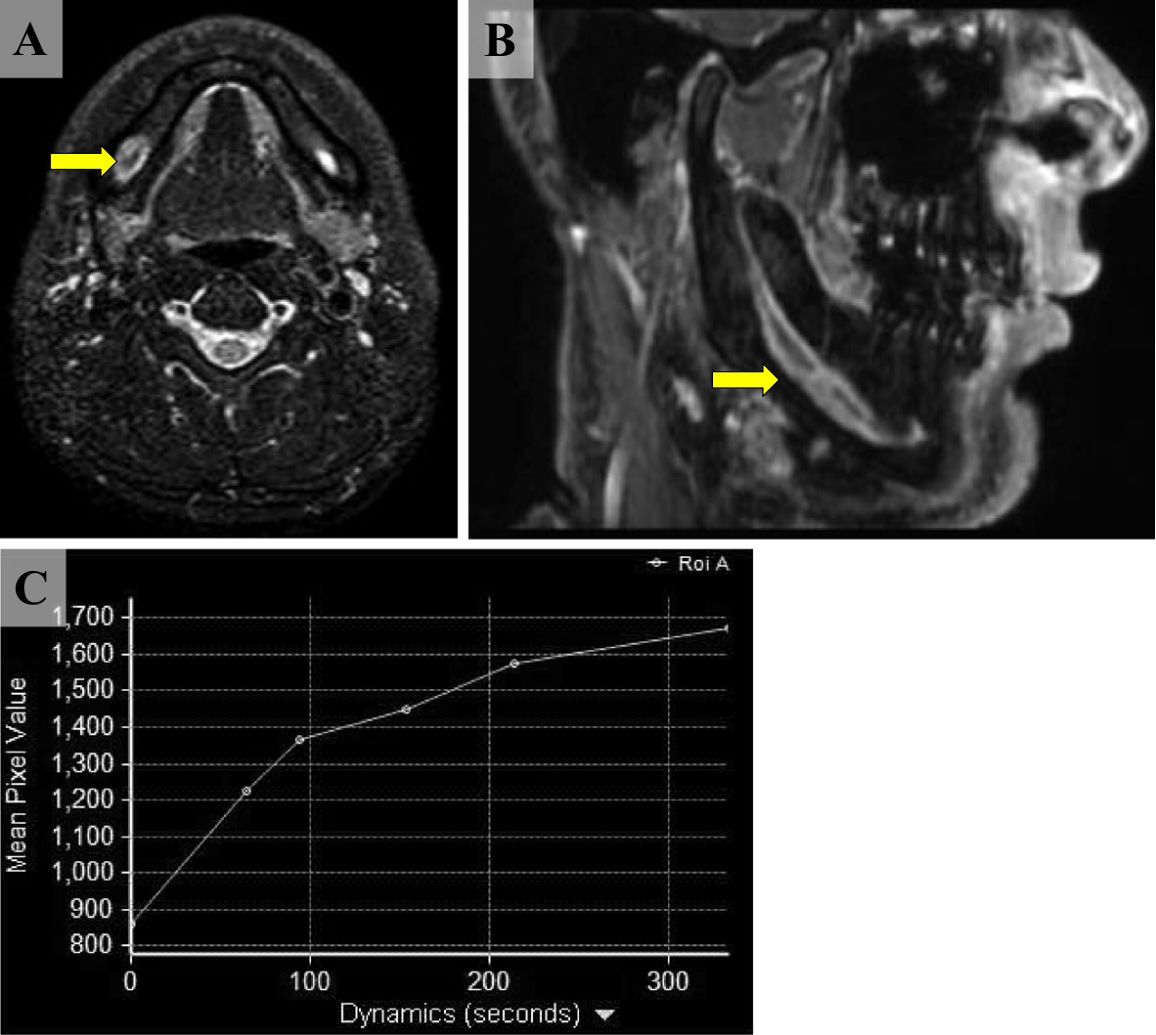
MRI findings. **a** Horizontal section. An irregular high signal in the STIR image was recognized (yellow arrow). **b** Sagittal sections. Dilation of the right mandibular canal was observed around the mandibular foramen (yellow arrow). **c** Dynamic images showed crescendo enhancement

His physical status was moderate and nutritional status favorable. There was no other notable factor. On visual inspection, there was no tumor lesion in the oral cavity (Fig. 1). Hypesthesia extending from the right lower lip to the mental region was noted, and the perception level was approximately 50% of that on the unaffected side. CT imaging revealed dilation of the mandibular canal (Fig. 2), and magnetic resonance imaging (MRI) showed an irregular high signal intensity on horizontal sections of short inversion time inversion recovery (STIR). On sagittal sections, dilation of the right mandibular canal was observed around the mandibular foramen. Dynamic images showed crescendo enhancement (Fig. 3).

**Fig. 4:**
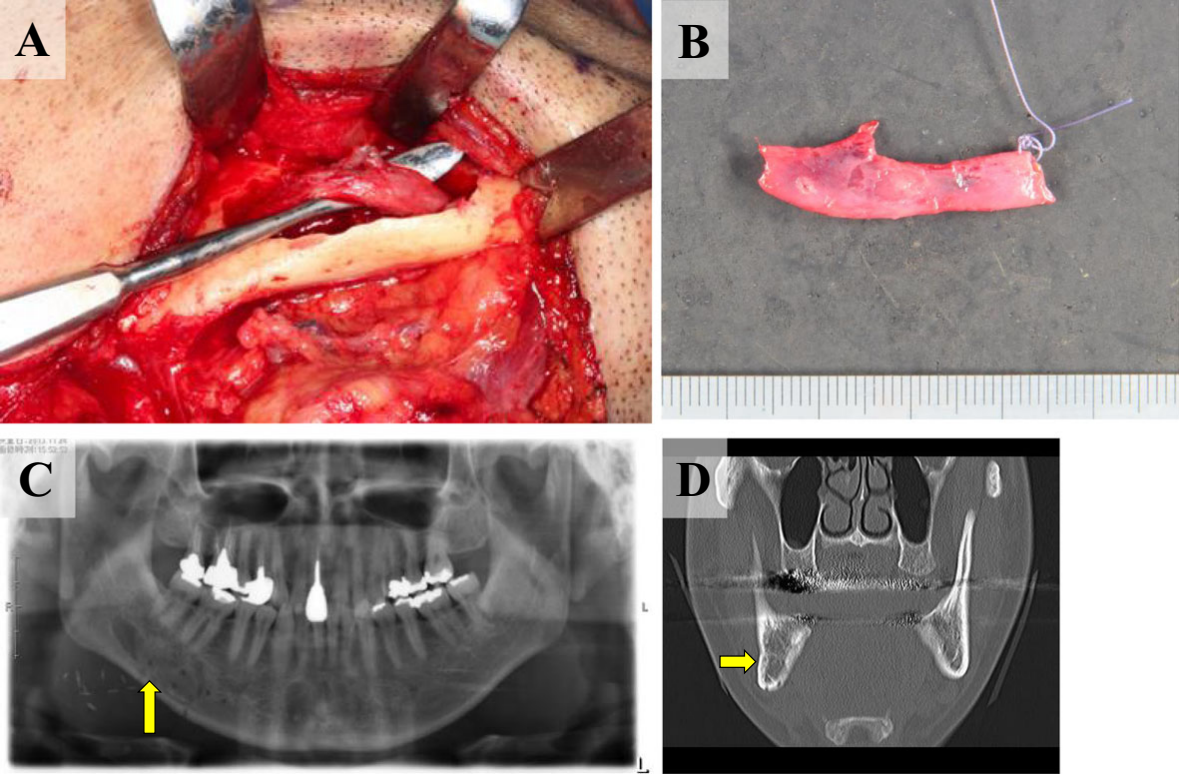
**a** Intraoperative findings. A cystic tumor of 3.2 × 1.0 cm was observed macroscopically following the inferior alveolar nerve. **b** Extirpation of tumor findings. The surrounding inferior alveolar neurovascular bundle was ligated and cut and removed as a lump. **c**, **d** Postoperative panoramic X-ray and X-ray CT findings. The radiopacity of the excised cavity was enhanced, and hyperostosis was confirmed

Preoperative biopsy findings carried out at the first hospital visited by the patient suggested a schwannoma involving the Antoni A region. Briefly, the lesion was immature, suggesting the presence of an active potential. Considering the risk of future tumor growth, the tumor was extirpated under general anesthesia in late April 2015. A macroscopically observed intraoperative finding was a cystic tumor (3.2 × 1.0 cm) associated with the inferior alveolar nerve and vessels; hence, the inferior alveolar nerve was ligated/cut and extirpated as a mass (Fig. 4a, b). Intraoperative rapid diagnosis confirmed the absence of tumor cells at the margins of the resected nerve specimen. During the 2-year postoperative follow-up, radiopacity was enhanced at the wound site after extirpation of the tumor, confirming favorable bone outgrowth (Fig. 4c, d). There have been no subsequent clinical findings or images suggestive of relapse.

**Fig. 5:**
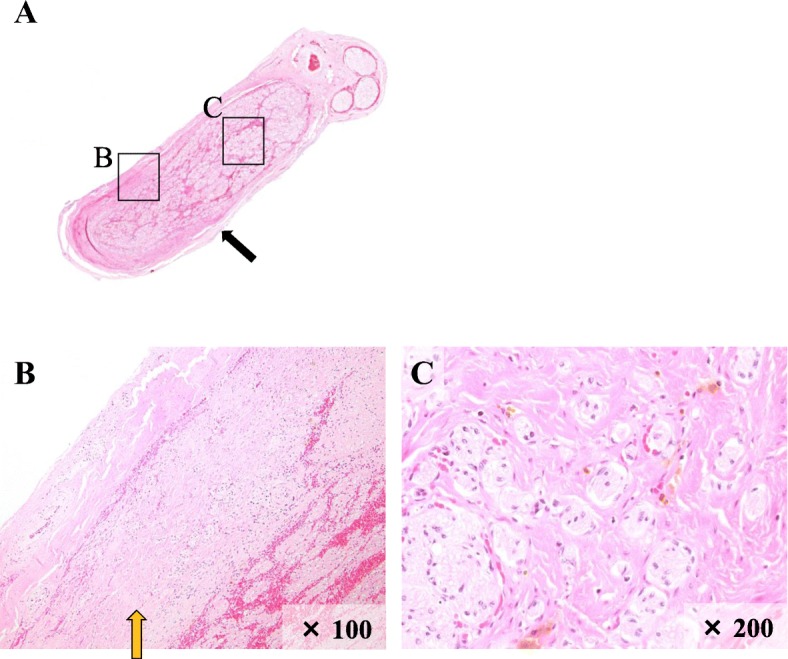
H-E staining. **a** Weak expansion. A tumor was found in the inferior alveolar nerve and inside (black arrow). **b** × 100. A thickened perineurium cell layer was confirmed (yellow arrow). **c** × 200. A part showing a pseudo-onion bulb-like structure confirming proliferation of tumor cells was observed so as to separate the nerve fiber bundle. B and C in Fig. 5a are enlargements of the same part as Fig. 5b, c

In a hematoxylin-and-eosin (H-E)-stained specimen, bundle-like tumor proliferation was observed around the nerve fiber. In addition, outgrowth of tumor cells was noted around nerve fibers, separating the nerve fiber bundle (Fig. 5a–c).

**Fig. 6:**
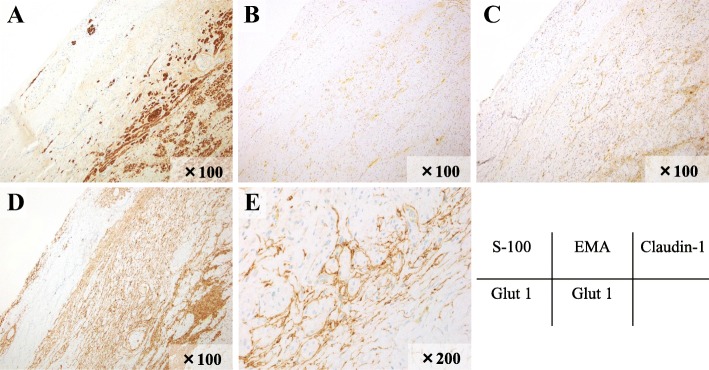
Immunohistochemical staining. The tumor cells were Glut-1 positive (**d**, **e**), weakly positive EMA (**b**), and Claudin-1 weakly positive (**c**). S-100 was negative for tumor cells (**a**), but positive for nerve fibers

**Fig. 7:**
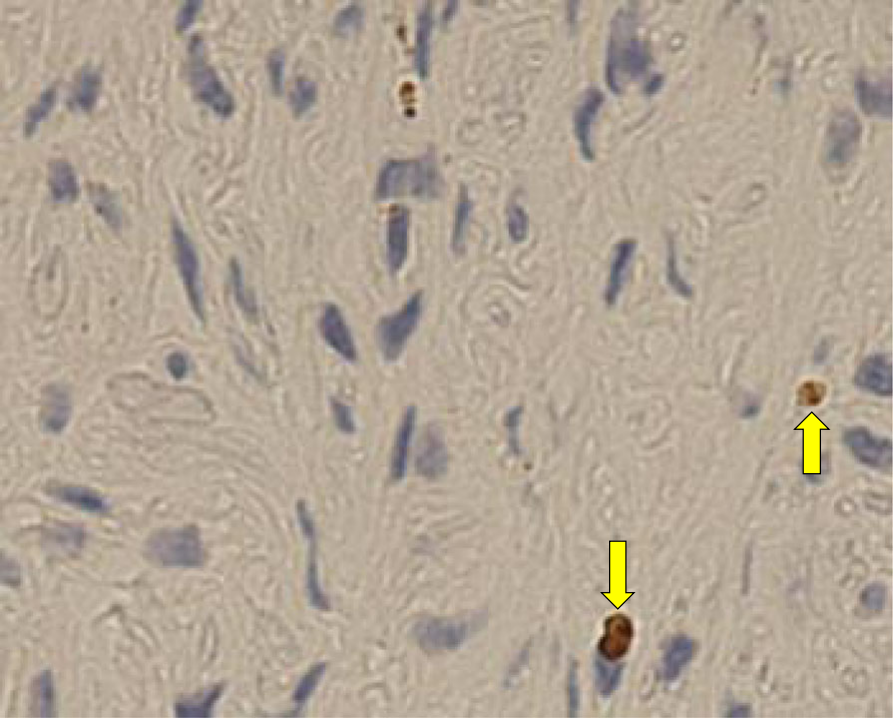
Expression rate of MIB-1 (Ki-67). The expression rate (%) of MIB-1 in INPN was 1.6% (positive nucleus: yellow arrow)

Immunohistologically, the tumor cells were positive for Glut-1 and weakly positive for EMA and Claudin-1. The tumor cells were negative for S-100, but the residual nerve fibers were positive for S-100 (Fig. 6a–e). Based on these findings, the histopathological diagnosis was INPN. The MIB-1 expression rate of INPN, indicating its cell proliferation ability, was 1.6% (Fig. 7).

**Fig. 8:**
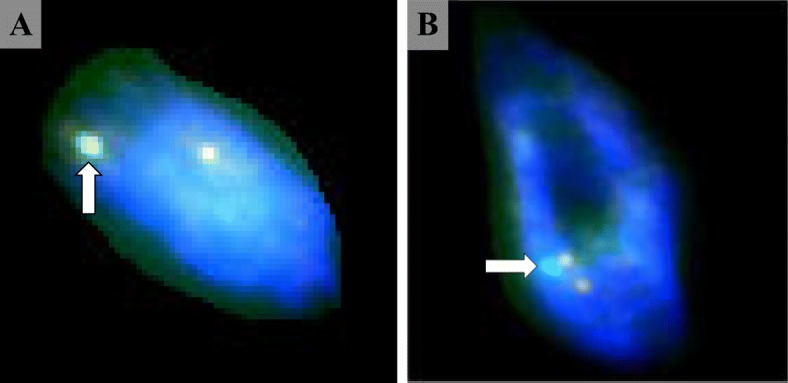
FISH (fluorescent in situ hybridization) method. **a** The BCR-ABL fusion gene shows a yellow signal. A yellow signal was observed, and expression of BCR-ABL fusion gene was confirmed (white arrow). **b** An orange signal indicates ABL gene and a green signal indicates BCR gene. Cells with one green signal of BCR gene were observed, and lack of BCR region was confirmed (white arrow)

To examine the pathogenesis of this tumor, FISH, which facilitates the detection of gene localization on the chromosomes, was carried out. In this procedure, a cloned gene or DNA fragment is labeled with a non-isotopic compound and hybridized with chromosomal DNA on a glass slide to directly detect the site of in situ hybridization, as a fluorescent signal, on a chromosome. FISH is safer and simpler than conventional autoradiography. In addition, it is advantageous as the results can be obtained in a short time. As standards, the fluorescent body of chromosome 9 (orange signal) indicates the ABL gene, that of chromosome 22 (green signal) indicates the BCR gene, and a yellow signal indicates the BCR-ABL fusion gene. Cellular signals on INPN were visually counted and classified. Measurement criteria for detecting the ALK fusion gene using the FISH method were quoted as diagnostic criteria. The principle is similar to that of BCR-ABL fusion gene detection. Samples with < 5 positive cells per 50 cells (< 5/50 or < 10%) were regarded as negative, and those with > 25 positive cells per 50 cells (> 25/50 or > 50%) were regarded as positive. The results showed that signals suggestive of BCR-ABL fusion gene formation were observed in 30 of 50 cells. In 12 of the 50 cells, the green signal of the BCR gene was present in the absence of fusion gene signals, suggesting deletion of the BCR region. Thus, 42 of the 50 cells were positive, suggesting chromosome 22 abnormalities. According to the above diagnostic criteria, our patient was regarded as having a chromosome 22 abnormality. Based on this finding, it was confirmed that this disease was a tumorous lesion (Fig. 8).

## Discussion

PN was first reported by Lazarus et al. in 1978 [4]. According to the WHO classification in 2013, it is defined as a “benign peripheral nerve sheath tumor consisting of perineurium cells.” PN is a peripheral nerve sheath tumor, but it is rare in comparison with schwannoma or neurofibroma. PN develops in the limbs, trunk, and head and neck region of adults. Most lesions are small, measuring ≤ 3 cm, although some become large. The sites of PN include the subcutaneous area, superficial layer of the dermis, and area deeper than the subcutaneous area. PN development in the oral cavity is very rare. Searching PubMed from 1981 until 2017 revealed 37 patients with oral PNs. Of these, 15 cases were INPN [2–14] including this present case (Table 1). Most reported oral INPN lesions were relatively small, and the mean diameter was approximately 2.1 cm (0.75–4.0 cm), including the present case. The most frequent site was the tongue in seven cases, next was mandibular in four including this present case, followed by the buccal mucosa in three, and lower lip in one.

**Table 1:** Clinical characteristics of oral intraneural perineuriomas (INPN)

Authors/year	Patient no.	Age	Sex	Location	Size (cm)	Treatment	Follow-up
Damm et al. 2003 [5]	1	26	F	Tongue	0.75	Resection	ND
Huguet et al. 2004 [1]	2	64	M	Mandible	2.0	Resection	ND
Da Cruz Perez et al. 2006 [6]	3	12	M	Tongue	0.6	Resection	6m NR
Siponen et al. 2007 [7]	4	18	F	Buccal mucosa	2.0	Resection	2y NR
Boyanton et al. 2007 [8]	5	6	F	Tongue	0.8	Resection	4m NR
Dundr et al. 2007 [9]	6	16	M	Buccal mucosa	1.5	Resection	2y NR
Kang et al. 2007 [10]	7	16	M	Tongue	10	Resection	6m NR
Tanaka et al. 2009 [11]	8	34	F	Tongue	0.6	Resection	8m NR
Vencio et al. 2009 [2]	9	59	F	Mandible	N/A	Resection	3y NR
Rocha et al. 2009 [12]	10	47	F	Tongue	1.0	Resection	1y NR
Rocha et al. 2009 [12]	11	37	M	Lower lip	0.5	Resection	1y NR
Hata et al. 2011 [3]	12	42	F	Mandible	1.8	Resection	6m NR
Hirano et al. 2013 [13]	13	14	M	Tongue	4.0	Resection	6m NR
Gomes da Silva et al. 2016 [14]	14	84	M	Lower buccal fold	2.0	Resection	5y8m NR
Present case 2018	15	43	M	Mandible	3.2	Resection	3y NR

*M* male, *F* female, *N/A* not available, *ND* no description, *NR* no recurrence, *y* year, *m* month

INPN involving the sensory nerve may cause sensory disturbance, and INPN involving the motor nerve may cause dyskinesia, such as progressive muscle weakness, but it rarely induces muscular atrophy. In the present case, hypesthesia involving the right lower lip extending to the mental region was observed.

In patients with mandibular PN, it is extremely important to evaluate the required extent of inferior alveolar nerve resection during surgery, as indicated for other neurogenic tumors. For evaluation, it is necessary to remove the cortical bone around the tumor, locate the tumor directly, and examine the state of adhesion to the inferior alveolar nerve or continuity with it. In previous studies, all cases, including the present case, underwent resection, and there was no report of relapse.

INPN is a nodular lesion. A large number of pseudo-onion bulb-like structures with a scroll-like proliferation of spindle cells around axons in the inner areas of nerves comprise the tumor parenchyma. In the present case, a solid proliferation of bundle-like spindle cells containing a large amount of cell components was observed around the inferior alveolar nerve. Partially, pseudo-onion bulb-like structures, as described above, were noted. Histopathologically, INPN was suggested.

Diseases to be differentiated from PN include schwannoma and traumatic neuroma. To make a definitive diagnosis, it is important to perform PN-specific immunostaining. Representative immunostaining methods primarily used for PN diagnosis include EMA, Claudin-1, and Glut-1. EMA is a high-molecular-weight membrane glycoprotein of epithelial cells. Patients with meningioma or peripheral nerve sheath tumors derived from perineurium cells show positive reactions to EMA. Claudin-1 is a tight-junction component protein, and it is utilized as a marker of the perineurium or its differentiation. Glut-1 is a member of the 12-pass transmembrane membrane protein family and is used as a perineurium cell marker. On the other hand, neuroglial and Schwann cells are positive for S-100 protein and negative for PN (Table 2). In the present case, tumor cells were positive for Glut-1/EMA and weakly positive for Claudin-1 on immunohistochemical staining. There were no S-100 protein-positive cells. Furthermore, H-E staining confirmed characteristic findings, leading to a definitive diagnosis of a perineurium cell-derived peripheral nerve sheath tumor, that is, INPN.

**Table 2:** Differential diagnosis of perineurioma (PN) in immunohistochemical staining (IHC)

Neurogenic tumor/IHC	EMA	S-100	Claudin-1	Glut-1
Perineurioma	+	−	+	+
Schwannoma	−	+	−	−
Neurofibroma	−	+	−	−
Traumatic neuroma	−	+	−	−

+ positive, − negative, *EMA* epithelial membrane antigen

Concerning the pathogenesis of PN, previous studies considered that localized hypertrophic neuropathy of perineurium cells in response to trauma, inflammation, and ischemia might be involved. However, recent studies reported the partial deletion of chromosome 22 (22q11) and chromosome 22 abnormalities [15, 16]. The hypothesis that PN may be a tumorous disease has been strongly supported, but whether it is a true neoplastic or reactive lesion has remained controversial. Previous studies reported the frequent expression of p53 and specific gene abnormalities in patients with PN. On the other hand, there were few phenomena, such as trauma or ischemia, in previously reported patients with PN despite the hypothesis that PN may be a reactive lesion. In recent years, it has been reported that chromosome 22 abnormality leading to the alteration of tumor suppressor genes that cause clonal proliferation of perineurial cells is a pathogenesis of its development in PN. In other words, cytogenetic changes and chromosomal abnormalities supporting the fact that PN is not a reactive change but a true benign tumor are thought to be the etiology [14].

Assuming that PN could be a tumorous disease, it is important to confirm the expression of cell growth markers. Proliferative cell nuclear antigen (PCNA), which is a nuclear protein that secondarily promotes DNA replication, and MIB-1 (Ki-67), which is a cell marker during cell growth in the G1, S, G2, and M phases of the cell cycle, were represented. The MIB-1 expression rate with PN involving the trunk ranges from approximately 4 to 14% [15]. However, based on the results of this survey, MIB-1 expression was detected in two patients with PN involving the oral and maxillofacial region, including the present case [17]. Accounting for ≤ 5%, the PCNA expression rate was 16.8% and the MIB-1 expression rate was 1.6% in the present case, suggesting proliferative capacity. This was within the range of expression rates in benign tumors. This activity is low, and the risk of relapse may be low if an adequate extent of resection is established.

FISH studies may clarify the process of tumorous proliferation related to PN-specific gene abnormalities. Previous studies confirmed the 22q11 deletion as a chromosome abnormality in patients with benign or malignant schwannoma, neurofibroma, or meningioma. Chromosome 22 deletion was confirmed in patients with peripheral nerve sheath tumors [15, 18, 19], and the long arm of chromosome 22 is known to involve a tumor suppressor gene, which may be involved in the pathogenesis of these diseases. Therefore, if the partial deletion of chromosome 22 (deletion of the long arm) is confirmed, it can be demonstrated that PN is a tumorous lesion, as reported for other nerve sheath tumors, due to the loss of a tumor suppressor gene. In a recent report, there is a pseudo-perineurioma (PPN) in the oral cavity caused by the proliferation of perineurial cells in the nerve [14]. This pathogenesis is thought to be associated with reactive lesions such as traumatic stimuli or conventional fibromas. Proliferation of this PPN resembles typical histological and immunohistochemical features in INPN, and it is reported that it also presents some common findings in its clinical features of INPN. It is said that INPN occurs in major thick nerves, whereas PPN occurs in a small branch of the nerve. PPN mainly occurs in the tongue and is said to be small in size as compared with INPN [14, 20]. However, at present, there is no clear diagnostic criterion between true PN and PPN. For the purpose of distinguishing, such as in this examination, cytogenetic search is considered necessary.

The BCR-ABL gene has been primarily confirmed in patients with chronic myeloid leukemia. If reciprocal translocation between chromosomes 9 and 22 occurs, a chromosome 22 that is shorter than the standard type, termed the Philadelphia (Ph) chromosome, may be formed. The c-ABL gene on the long arm of chromosome 9 and the BCR gene on the long arm of chromosome 22 are fused to the Ph chromosome, forming the BCR-ABL gene. BCR-ABL gene-coded BCR-ABL thyrosine kinase is produced, and its activation induces neoplastic transformation of cells [21]. In the present case, a chromosome 22 aberration and BCR-ABL gene expression were confirmed by fluorescence in situ hybridization (FISH), thereby verifying that PN is a tumorous disease.

## Conclusion

We encountered a patient with mandibular INPN and a chromosome 22 aberration. The results of examination using the FISH method demonstrated that INPN is a tumorous disease. This is the first report on BCR-ABL fusion gene expression in the presence of INPN.

## Abbreviations

ENPN: Extraneural perineurioma
FISH: Fluorescent in situ hybridization
INPN: Intraneural perineurioma
PCNA: Proliferative cell nuclear antigen
PCs: Perineurial cells
PN: Perineurioma
